# DNA nanostructures: A versatile lab-bench for interrogating biological reactions

**DOI:** 10.1016/j.csbj.2019.06.013

**Published:** 2019-06-14

**Authors:** Andrew J. Lee, Christoph Wälti

**Affiliations:** Bioelectronics, The Pollard Institute, School of Electronic & Electrical Engineering, University of Leeds, LS2 9JT, United Kingdom

## Abstract

At its inception DNA nanotechnology was conceived as a tool for spatially arranging biological molecules in a programmable and deterministic way to improve their interrogation. To date, DNA nanotechnology has provided a versatile toolset of nanostructures and functional devices to augment traditional single molecule investigation approaches – including atomic force microscopy – by isolating, arranging and contextualising biological systems at the single molecule level. This review explores the state-of-the-art of DNA-based nanoscale tools employed to enhance and tune the interrogation of biological reactions, the study of spatially distributed pathways, the visualisation of enzyme interactions, the application and detection of forces to biological systems, and biosensing platforms.

## 1 Introduction

The desire to decipher and understand the form and function of biological macromolecules, as well as the mechanisms of the many interaction networks that are fundamental to the machinery of all living organisms has yielded significant scientific activity in this field for well over a century. A more in-depth understanding would empower us to harness, engineer and repair biological systems to produce sustainable fuels, enhance food production, diagnose and treat disease, *inter alia*. To date, the breadth of our understanding is mainly derived from techniques which analyse large ensembles of identical molecules and systems concurrently. These include a plethora of biochemical, structural, and molecular biology approaches which, when coupled with biophysical analysis, have provided significant insight into the form, function, and kinetics of a vast array of biological systems. However, these methods only report averaged information. This can be seen most keenly in cryo-electron microscopy (cryoEM) which, although it provides unparalleled atomic-level structural detail of biomolecules, it does generally require an ensemble of >100,000 instances of the subject macromolecule to provide such detailed information. As a result, these approaches are insensitive to the often not readily visible heterogeneities of complex biological systems, which are arguably of significant importance.

While sophisticated ensemble-average studies will undoubtedly play a major role in furthering our understanding of biological systems, it is also clear that techniques which enable single-molecule studies are highly desirable and will provide important information that is often complementary to that obtained from broader studies. Significant advances in single-molecule techniques have been reported over recent decades, and depending on the desired quantity to be studied, time-resolved single-molecule FRET, [1] mechanical measurements using magnetic [2] or optical [3] tweezers and traps, and direct visualisation with atomic force microscopy [4,5] go some way to achieving this. Moreover, techniques which attempt to aspirate or inject single molecules from biological systems such as cells, including nanopipettes, [6] dielectrophoretic trapping with nano-electrodes [7] or the use of nanopores [8] can offer routes to single entity quantification. However, despite the rapid advances in these techniques, substantial challenges in isolating, manipulating, and in particular contextualising discrete biological entities for the purposes of these studies remain, which limits their utility.

**Fig. 1:**
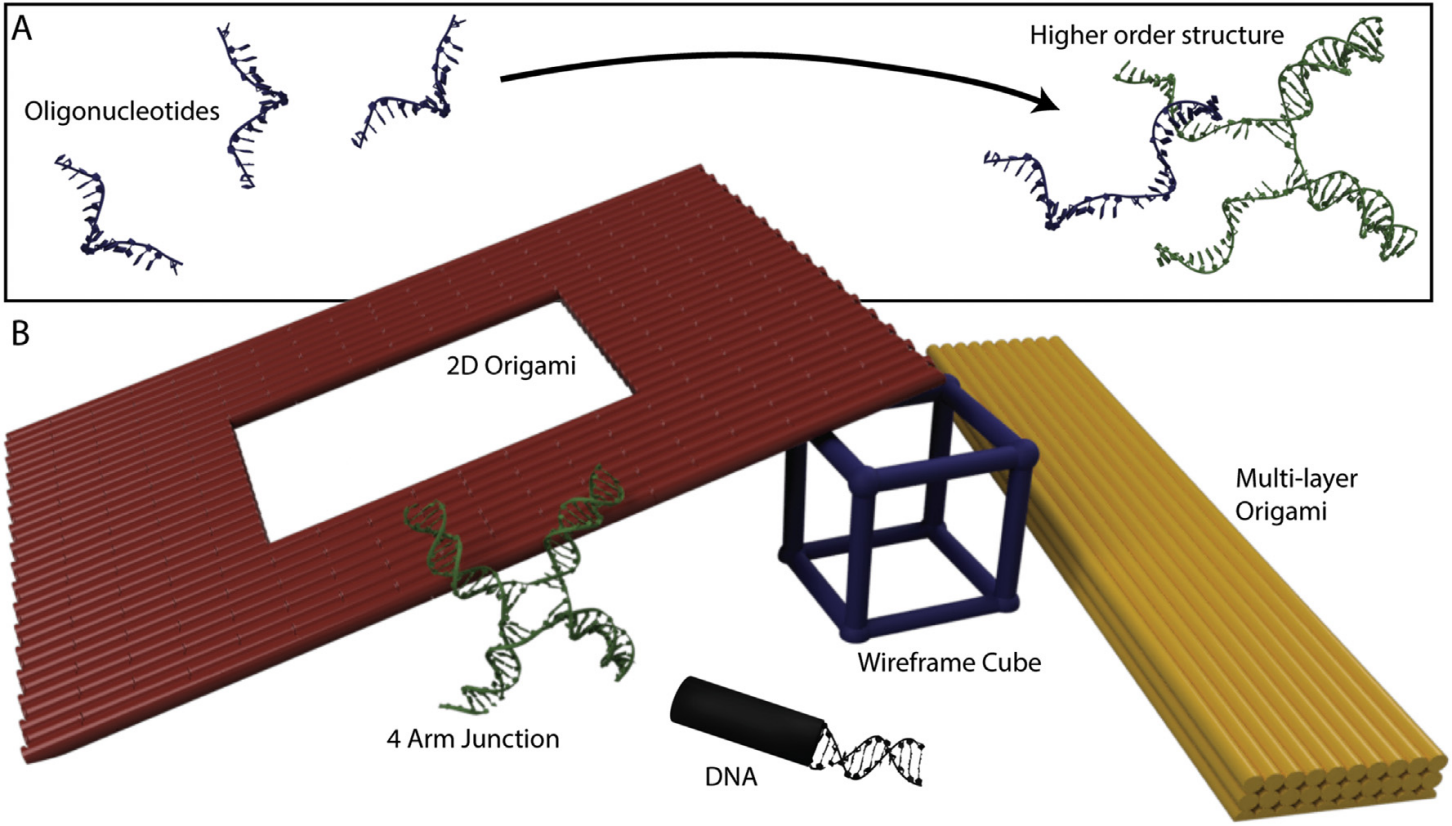
Constructing with DNA. A) A schematic diagram depicting the construction paradigm central to all non-canonical DNA-based nanostructures. B) Examples of architectural forms of DNA nanostructures, including multi-arm branched junctions, wire-frame structures, DNA origami and 3D multi-layered objects.

An emerging solution to this challenge is the use of DNA-based nanostructures to arrange discrete biological entities in a highly programmable manner, providing a spatial connection between the readily accessible micro environment and the biological system at the nanoscale. The field of DNA nanotechnology (Fig. 1) has grown significantly over recent decades owing to the versatility of its intrinsic lock-and-key assembly mechanisms and the abundance of commercial nucleic acid synthesis.

DNA nanotechnology – originally inspired by the native Holliday junctions (Fig. 1) – harnesses DNA as a structural polymer, making use of the high-fidelity base-pair interactions to assemble different single-stranded DNA molecules into complex shapes. Collections of nucleic acids can form pre-designed non-canonical structures whose arrangements are specified by their base-pairing. However, in 2006 DNA nanotechnology expanded well beyond the design of multi-arm branched arrangements and their derived wireframe structures with the arrival of the highly efficient scaffolded design paradigms, known as DNA origami [9]. Importantly, the well-defined base-pairing provides a simple but powerful language for creating internally-programmed self-assembling designs, arguably one of the main reasons for the success of the field [10].

Notable advances in the DNA origami approach have taken the field from two-dimensional discrete and planar lattice structures [11,12] to three-dimensional objects, encompassing, for example, solid, [13] hollow and wireframe designs (Fig. 1B) [[14], [15], [16]]. Moreover, current design strategies allow for flexible and responsive elements to be incorporated to enable programmable action [17]. In conjunction with these sophisticated structural designs, a vast array of nucleic acid chemical modifications are available to enhance these nanostructures, thus enabling the spatial organisation of a multitude of molecular-scale components from individual chemical groups [18] to peptides and proteins, [[19], [20], [21], [22], [23]] and even inorganic objects such as carbon nanotubes [24] or gold nanoparticles [25,26]. With this in mind, it is clear that DNA nanotechnology can provide the foundations for unique and adaptable tools capable of localizing and organising discrete biological entities. Furthermore, DNA nanotechnology finds applications in clinical therapeutics, with a focus on their potential as functional drug delivery systems *in vivo* [27].

This review aims to provide an overview of the application of DNA-based nanostructures in the static as well as dynamic analysis and characterisation of functional biological entities and their complex pathways at the single molecule level.

## 2 Spatially Defined Reactions

**Fig. 2:**
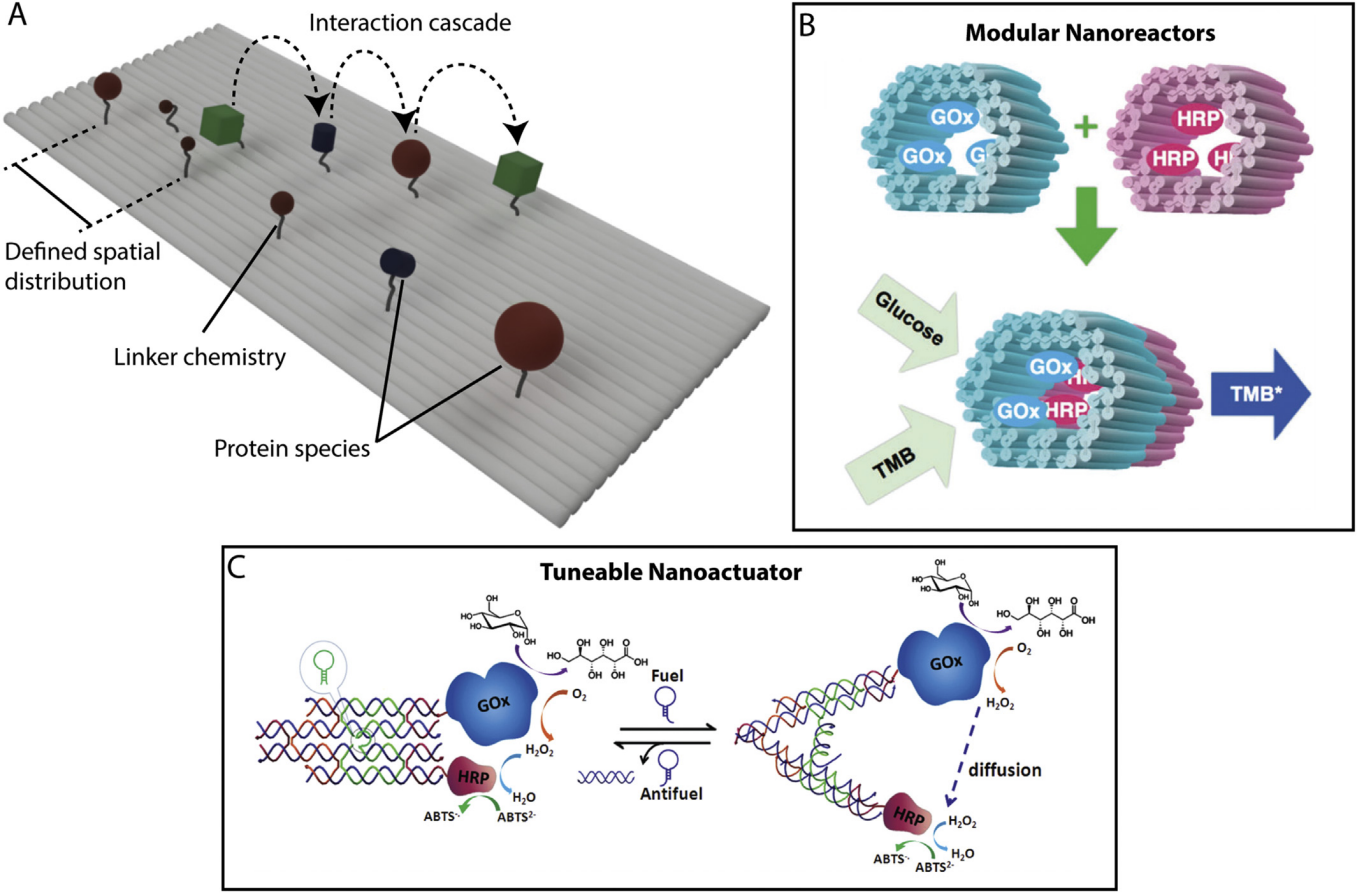
Spatially distributed enzyme systems. A) Schematic diagram depicting a recapitulated enzyme cascade on the surface of a DNA nanostructure. B & C) Enhanced reaction rates can be achieved through the confinement of reacting species within modular DNA-based nanoreactors (B) or tuned dynamically using nanoactuators (C). Panels B) and C) are adapted with permission from Linko et al [28] copyright: (2015) The Royal Society of Chemistry, 2015 and Xin et al [29] copyright: (2013) John Wiley and Sons, respectively.

Biological functionality is mediated through interacting species in specific pathways by either a defined temporal or spatial organisation. Within a native context this is largely achieved through compartmentalisation or association of species at or within membranes. Alternatively, DNA nanostructures provide a controllable mimic, able to define the spatial distribution, relative orientation and stoichiometric relationships of biological entities with sub-nanometre resolutions. Arguably, DNA nanostructures may be considered as molecular pin boards on which the entities of interest can be localised for study (Fig. 2).

Early examples of this approach by Voigt et al. demonstrated direct coordination of chemical reactions taking place on the surface of DNA nanostructures [18]. Although these studies were not strictly biological in nature, they provide an excellent example of studying spatially defined processes at the single molecule level using DNA nanostructures. In order to visualise directly the active chemistry the authors used atomic force microscopy (AFM), and the chemical groups were sandwiched in-between the DNA nanostructure and a fiducial biotin/streptavidin marker. Using this approach, the authors demonstrated the formation of specific patterns through the selective breakage or formation of a variety of chemistries, including the reduction of disulphide bonds, singlet oxygen nucleophilic attack, and the formation of triazole and amide groups from the reaction of azide and alkyne, and ester and amine groups, respectively. Helmig *et al* [30] adapted this approach to report on the diffusion of singlet oxygen from an immobilised indium pyrophenophorbide photosensitiser to the surrounding array of liable linker substrates on the surface of a DNA nanostructure. This work highlights that DNA nanostructures provide uniquely addressable platforms for probing the spatial relationships between reactive species, appealing to the study of coupled enzyme systems.

Where enzymes act as part of a coordinated network or cascade, the diffusion of reactive species is often a limiting factor in the efficiency of the overall system. DNA nanostructures can provide a unique approach to control precisely the spatial and stoichiometric relationships between different constituent enzymes (Fig. 2A). This can be orchestrated through the arrangement of 2D arrays, such as those used to study the parts of the Xylose metabolic pathway [31] or the Pentose phosphate pathway [32]. Or by designing nanostructures for the confinement of reactive species within 3D containers, so called nanoreactors (Fig. 2B), which have been demonstrated to enhance the efficiency of coupled enzyme systems [28].

The quintessential example of a coupled enzyme reaction is the glucose oxidase (GOx) – Horseradish Peroxidase (HRP) system which was used as a reporter in early blood glucose sensing applications. GOx oxidises glucose to form gluconolacetone, and in the presence of oxygen the enzyme is then reoxised and H_2_O_2_ is produced as a byproduct. H_2_O_2_ is subsequently oxidised by HRP which yields two electrons which lead to a measurable current. Thus the activity of the enzyme pair (and hence the glucose concentration) can be determined *via* the resulting current [33].

Although this system operates natively in solution, to date there have been a host of DNA-bound enhanced GOx/HRP cascades demonstrated, enabling exploration and optimisation of the spatial dependence and reactive species confinement of this enzyme couple. These include arrangements of GOx/HRP on linear single-stranded DNA (ssDNA), [34] linear double-stranded DNA (dsDNA), [35] Hexagonal DNA lattices, [36] 2D DNA origami, [37] and the confinement of reactive species within DNA tubes and DNA nanocages [28,38,39]. Of particular note are the related works of Xin *et al* [29] and Xing *et al* [40] which independently demonstrated tuneable efficiencies of this system – with up to 1.5 times enhancement compared to the solution reactions – through the use of tweezer-like nano-actuators (Fig. 2C). Here, DNA strand displacement is used to modulate the nano-actuators between open and closed states, specifically altering the separation distance between the tethered enzymes. Although previous works have explored the precise spatial relationship between this enzyme pair, these studies highlight the possibility of tuning their efficiency *in situ* and on demand. More recently, the Andersen group have combined these actuation and confinement approaches together to create an impermeable nanovault capable of encapsulating the endopeptidase enzyme alpha-Chymotrypsin (aCt). When the DNA-based container was closed, the enzyme was shown to be sequestered away from its substrate within the same environment and thus its activity could be modulated by repeatedly opening and closing the container using a strand displacement locking mechanism [41]. Such approaches to modulating and remotely activating or deactivating enzyme systems could have far reaching potential in diagnostic and industrial applications, where targeted or timed responses are desired. For a comprehensive discussion of the field, we refer to the review by Rajendran et al. [42].

## 3 Direct Visualisation of Biomolecular Processes

In contrast to systems that require well defined spatial relationships – such as signal transduction cascades – the function of many biological entities are less tightly spatially constrained relative to their substrates and these systems must undergo free movement in order to perform their function. Therefore, it would not be appropriate to tether these systems to a DNA nanostructure for studying, as tethering would significantly impair their function. Alternatively, DNA nanostructures can be used to host reaction substrates in order to localise the enzyme activity within a pre-defined spatial window for interrogation, for example by high speed AFM (HS-AFM) or other appropriate tools.

**Fig. 3:**
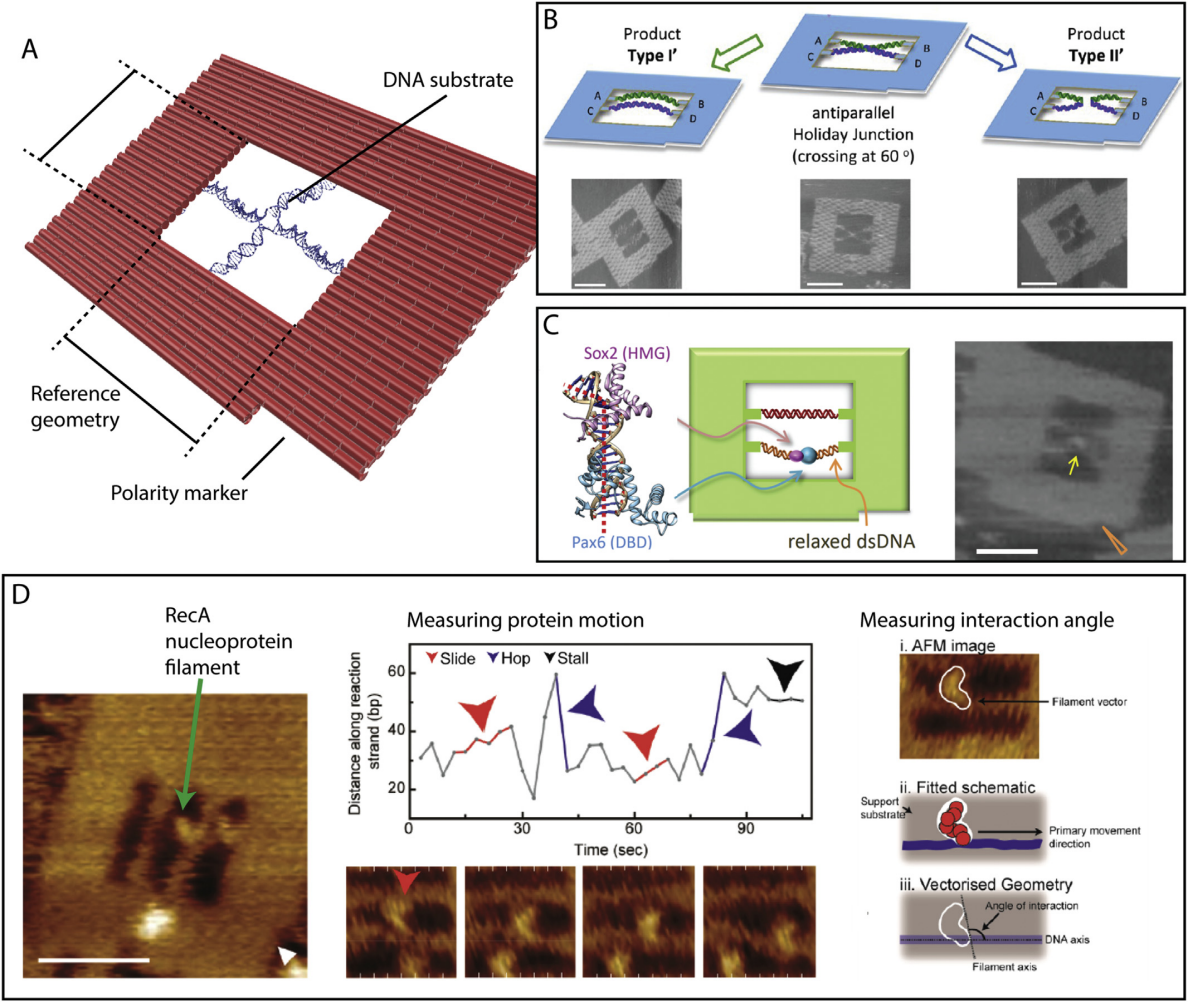
Contextualising biomolecular interactions directly at the nanoscale. A) A schematic diagram depicting a DNA origami frame-like structure containing a complex topological DNA substrate for observing enzyme activity with HS-AFM. The structure provides a support for the substrate whilst contextualising the ensuing enzyme reaction with a known reference geometry and orientation. B – D) This approach enables enzyme binding preference (B), DNA topological change (C) and specific interaction dynamics (D) to be directly interrogated at the single molecule level. B, C and D are adapted with permission from Yamamoto et al [56] copyright: (2014) American Chemical Society, Suzuki et al [54] copyright: (2014) American Chemical Society and Lee et al, [59] copyright: (2018) American Chemical Society, respectively. All scale bars = 40 nm.

Endo *et al* employed a DNA origami frame-like structure (Fig. 3A) capable of hosting tandem dsDNA molecules to investigate how *Eco*RI methyltransferase was regulated by the conformational flexibility of its substrate, *i.e.* the tandem dsDNA [43]. In this work, one DNA substrate was placed under tension while the second one was left slack and thus more flexible. The two dsDNA substrates were arranged parallel within a cavity at the centre of a DNA origami structure. Extraordinarily, not only were the authors able to observe the binding of EcoRI methytransferase in real time using HS-AFM, but they were able to discriminate the binding preference of the enzyme to the flexible DNA strand and measure the induced bend angle of the DNA once bound [43]. Following this, the approach was subsequently expanded to study several DNA topological transitions, including B – Z form DNA transitions, [44,45] G quadruplex formations, [[46], [47], [48], [49], [50]] and the action of a Zn^2^-dependent DNAzyme. [51]

The use of DNA frame structures to host the biological reaction systems for AFM observations of their activity has many benefits over previous work where the biological systems were largely unconstrained. In particular the ability to contextualise the interaction species within a known geometry – given the defined dimensions of the DNA origami structure and the typical inclusion of a polarity maker to identify orientation (Fig. 3A) – offers many benefits. Furthermore, inbuilt control substrates can be provided and the inclusion of functional chemistry, such as photocleavable linkers, can enable parts of a reaction to be initiated through external means.

The known position and orientational geometry of the observed reaction enables the determination of characteristics such as the arrangement and angles of specific topological states of DNA formed as the result of enzyme activity. These can be discerned and statistically analysed, for example the different dsDNA products of the RecU, Muc1 or Cre recombinase [[52], [53], [54]] resolution of Holliday junctions (Fig. 3B) have been reported. In addition, with the inclusion of parallel arranged substrates – in some cases used as a control – the statistical analysis of the binding or functional preferences of enzymes as a result of sequence (*e.g.* a specific binding site) or DNA modification can be achieved (Fig. 3C) [[55], [56], [57]]. Moreover, the DNA structure itself can be used as a measurement standard against which the position of an interaction species can be measured over time, for example, this approach was used to measure the progress and derive a rate estimate of T7 polymerase RNA transcription *in vitro* [58]. It is, however, important to be mindful of the limitations of the microscopy system when attempting to define these quantities, in particular the temporal resolution and the challenges caused by the confinement of species to a surface.

Our recent work has exploited these advances to decipher the homology searching mechanism of the ubiquitous recombination enzyme RecA (Fig. 3D) [59]. RecA forms a nucleoprotein polymer which locates regions of DNA that share sequence homology in the preliminary steps of a strand exchange reaction that is central to DNA repair. Conducting the homology searching experiments within a DNA origami cavity we directly observed the previously debated involvement of facilitated diffusion of the RecA nucleoprotein complex along the dsDNA substrate, deconvoluting the small registration steps from the larger scale random sampling interactions taken by the complex. Moreover, as a result of the known substrate geometry we were able to identify the angle of interaction (106°) of the nucleoprotein complex and DNA substrate and thus confirm that the binding interaction takes place within the proposed secondary binding pocket on the external surface of the RecA structure.

The list of biological systems studied with these tools to date is by no means exhaustive and there remains a large number of important mechanisms and pathways that are not well understood as yet and direct investigations using this approach would arguably contribute to furthering the understanding. In particular, our current work aims to expand this approach from DNA-based enzymatic reactions to those which involve protein–protein interactions, and to address recruitment of enzymes into multicomponent complexes – such as the DNA transcription complex.

## 4 Super Resolution Optical Rulers

**Fig. 4:**
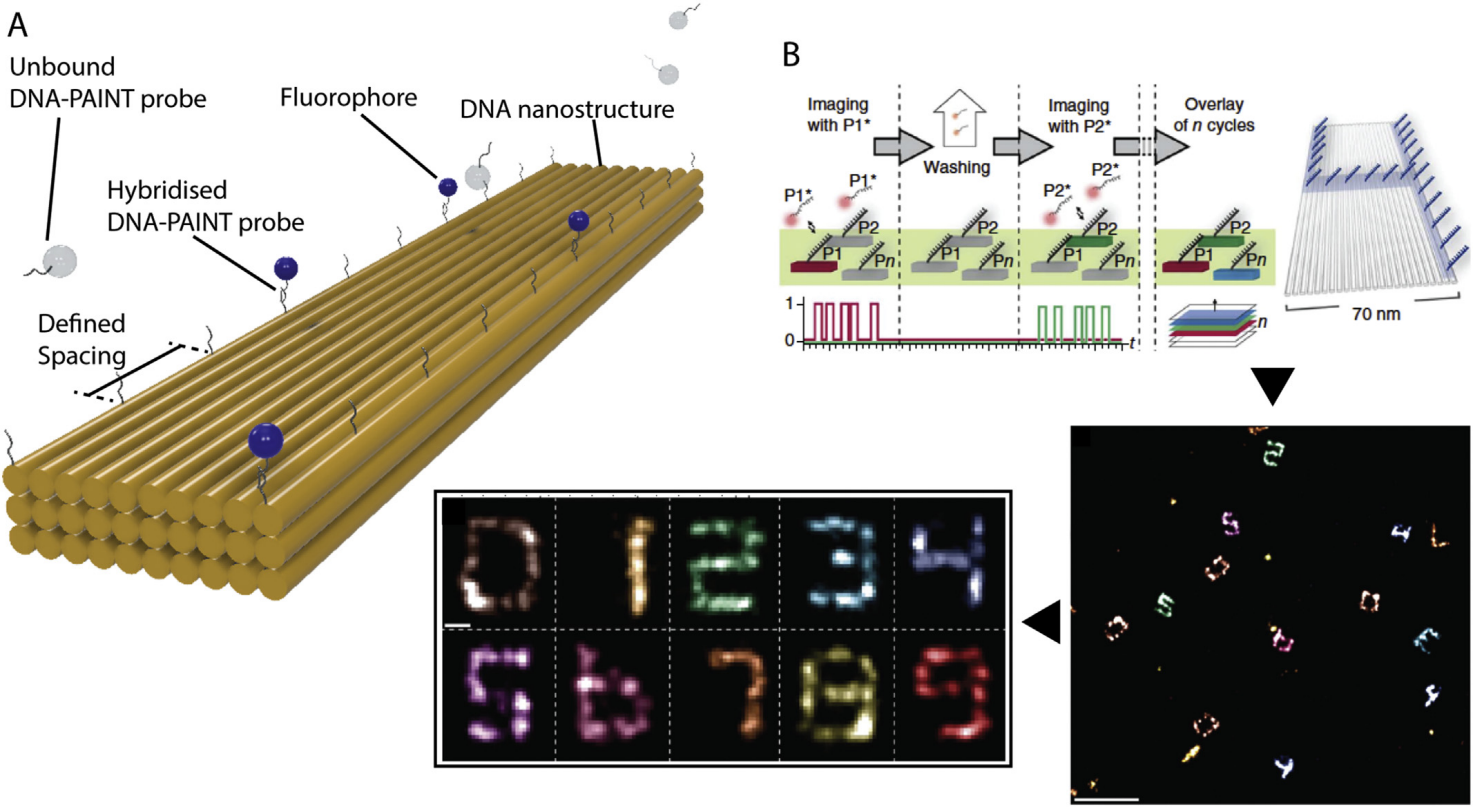
DNA-based optical super resolution calibration references. A) A schematic diagram depicting the assembly of fluorophores with a defined spatial distribution on a rigid DNA nanostructure, indicated for the DNA-PAINT imaging regime. B) The sequential washing and imaging steps of the Exchange-PAINT approach which enables multiplexed imaging with the same fluorophores attached to different DNA hybridisation probes within the same sample. Here, the digits 0–9 are demonstrated in false colour on the surface of a 70 nm DNA nanostructure through successive imaging cycles. B Is adapted with permission from Jungmann et al [61] copyright: (2014) Springer Nature.

Over the last decade, DNA nanostructures have found an additional application in augmenting optical microscopy to provide *in situ* calibration standards for a host of single molecule localisation super resolution imaging approaches (Fig. 4). This is due, in part, to the flexibility and addressability of the DNA nanostructures themselves and the commercial availability of fluorescently labelled nucleic acids. The DNA origami approach has been used to produce nanoscopic calibration standards for super resolution techniques including stochastic reconstruction microscopy (STORM) and photoactivated localization microscopy (PALM) (Fig. 4B) [60]. Both PALM and STORM are able to circumvent the classical diffraction limit of light microscopy by temporally activating or “switching” the fluorophores sequentially and thereby localizing the emission of one dye at any given time point. Where this is performed for two dyes separated by less than the diffraction limit, the two localisations can nevertheless be spatially deconvoluted, with achievable resolutions of a few tens of nanometres. The localisation of the dyes with a known spacing on a DNA nanostructure makes them the ideal standard against which to calibrate these approaches.

Furthermore, the well characterised thermodynamics of DNA hybridisation has lent itself to additional advances in super resolution microscopy in the form of DNA-mediated Points Accumulation In Nanoscale Topography (DNA – PAINT) imaging. This approach, developed by Jungmann et al. [62]. adapted the previously reported PAINT technique by using short ssDNA sequences to encode the specific, yet transient, binding of dyes to a target.

Instead of chemically switching fluorophores localised at fixed locations, the stochastic switching behaviour is achieved through transient hybridisation of a fluorophore-containing short DNA oligonucleotide – or imager strand – and another short DNA oligonucleotide – or docking strand – which is fixed to the target (Fig. 4A). By using simple oblique illumination [63] or total internal reflection only the fluorophores transiently localised within the imaging plane are observed as blinking points with the unbound imaging strands contributing little background to the image whilst diffusing freely in bulk solution [62,64,65].

This has circumvented many of the limitations in the multiplexing of other stochastic switching techniques, such as STORM and PALM, by removing the need to re-optimise buffer conditions for different fluorophores. Thus, the multiplexing of DNA-PAINT is simply achieved for spectrally separated dyes linked to orthogonal DNA sequences simultaneously imaged within the same sample. Alternatively, more advanced multiplexing can now be achieved using simplified laser setups within the same sample through sequential use of the same dye attached to orthogonal DNA sequences (referred to as exchange PAINT) (Fig. 4B) [61] or by target-correlated FRET efficiency differentiation (referred to as FRET PAINT) [66]. This use of FRET pairs spatially organised by DNA builds on conceptually similar work of the conformational dynamics of DNA hairpins [67,68] and Holliday junctions [69] by others. Interestingly, these latter exchange-based methods have more recently been adapted to multiplex STORM approaches, by sequentially cycling through transient and stable DNA hybridisation environments for a set of different target probes [70].

DNA-PAINT has now evolved to provide true quantitative measurements, which has been demonstrated for a number of individual proteins *in vivo* [71], and to quantify the incorporation efficiencies of oligonucleotides within DNA origami substrates *in vitro* [65]. More recently, proponents of the technique have moved away from the traditional and expensive use of DNA labelled antibodies towards DNA-based aptamers [72] and peptide-based antibody mimetics [73] for target binding, increasing the versatility and accessibility of the technique. As such, DNA nanotechnology is now enabling optical microscopy to be pushed further than ever before, bringing the quantification of single biological entities into context within a native cellular environment.

## 5 Studies of Biomechanics and Interaction Forces

**Fig. 5:**
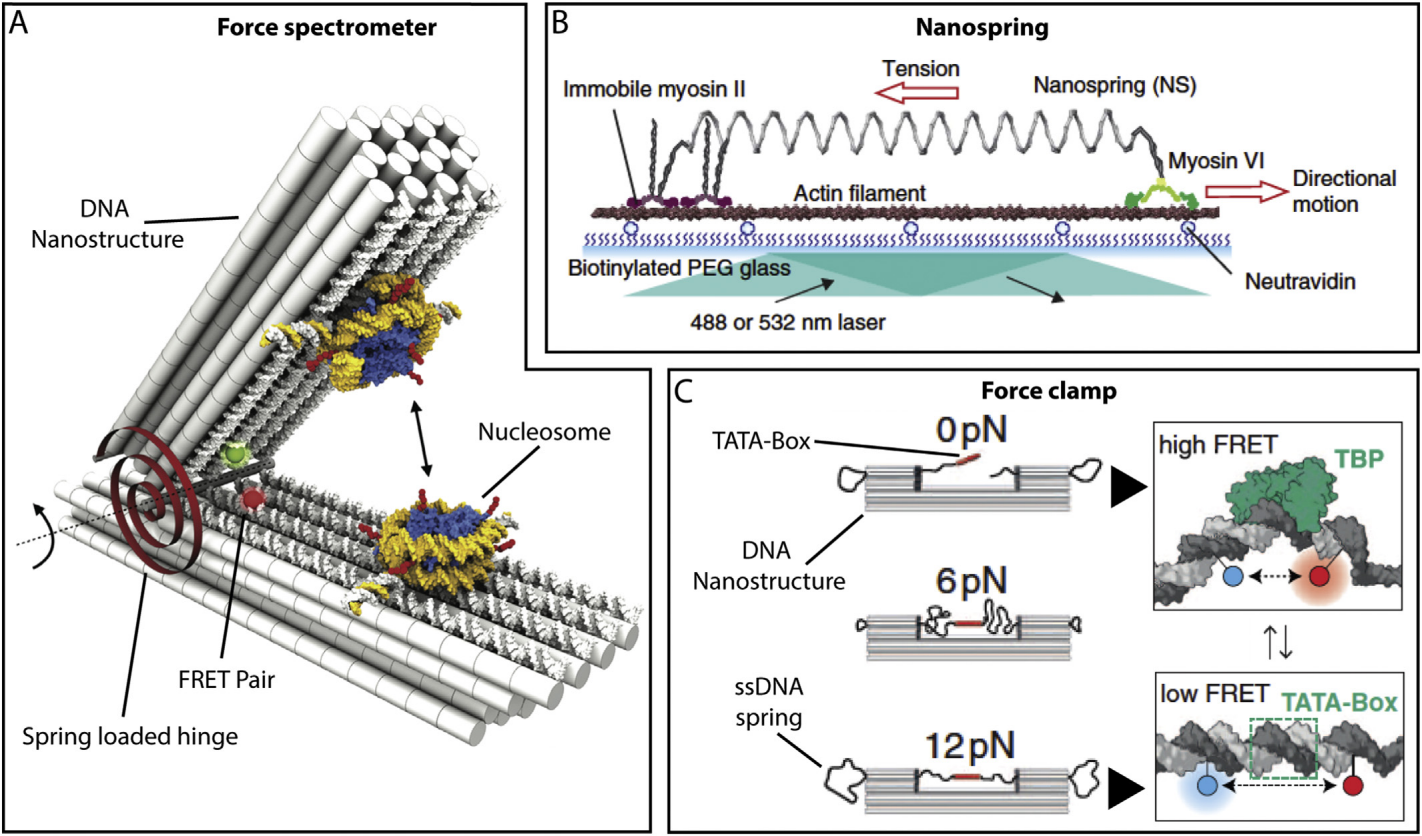
Generating and sampling forces with DNA nanostructures. DNA devices, including tweezer-like systems (A), entropic springs (B) and clamps (C) have been used to apply resistive forces in order to enable force-investigations at the single molecule level. These systems have been used to measure the interaction forces between nucleosomes (A), the pulling force generated by Myosin VI (B) and the mechanosensitive binding of enzymes to DNA (C). A, B and C are adapted with permission from Funke et al [74] copyright: (2016) American Association for the Advancement of Science, Iwaki et al [75] copyright: (2016) Springer Nature and Nickels et al [76] copyright: (2016) American Association for the Advancement of Science, respectively.

DNA nanostructures cannot only be used as static support structures but can be designed as functional actuators capable of generating and sampling forces between individual biological systems with exquisite detail. An excellent example of this was contributed by the group of Hendrik Dietz [74] where a set of spring-loaded nano-tweezers was employed to measure the energy landscape between pairs of interacting nucleosomes in order to gain better insight into the action of chromatin compaction of the genome (Fig. 5A).

Moreover, DNA based devices have been used to impose defined resistive forces to biological entities such that the mechanosensitivity of the system can be probed. Recent work from Shih and co-workers developed a tunable nano-spring to probe the effect of applied resistive force to the binding and walking mechanism of Myosin VI (Fig. 5B) [75]. The nano-spring was formed from a two-helix bundle with a negative superhelical strain, the spring constant of which could be controlled by the extent of the superhelical turns. This enabled the authors to detect a force-dependent transition in the walking mechanism of Myosin VI, and the same approach could be applied to studying the mechanosensitivity and generated force of other molecular motors.

Using a conceptually similar approach, the Liedl group [76] has provided a nanoscale forceclamp for studying the mechanosensitivty of gene regulation, using the suppression of DNA bending, and therefore reducing association, of the TATA-binding protein (Fig. 5C). Here, a ssDNA molecule acted like an entropic spring when held within the confines of a DNA origami clamp. The applied tension on the dsDNA TATA-binding element at the centre could be controlled in the low piconewton range, by loosening or tightening the ssDNA slack in the design. The authors found that disruption of the binding occurred when forces in excess of 10 pN were employed.

## 6 Biosensing and Assays

Another promising use of DNA nanostructures is as carriers for biosensing or localised biological assays. One of the earliest examples of this was the multiplexed RNA detection system demonstrated by Ke et al. [77]. The authors constructed a label free assay for detecting specific RNA sequences corresponding to the *c-myc*, *Rag-1* and *beta-actin* genes through hybridisation with DNA probes protruding from the surface of a DNA nanostructure. Once bound, the RNA – DNA pair formed a rigid structure, readily identifiable by AFM when compared to the ssDNA probes alone. A Similar approach was taken by Seeman et al. [78]. in developing a DNA nanochip for single nucleotide polymorphism (SNP) detection. Here, DNA probes were arranged on the nanostructure to spell the letters A, T, G and C – each contained the corresponding SNP of interest within the target sequence. Detection was achieved through isothermal branch migration with the target sequence initiated at a small toehold on the probe. This led to the removal of the specific probe and consequently the loss of the corresponding letter was visualised on the DNA nanostructure.

Alternative work by the Bald group has produce spatially defined arrays to assay quantitatively UV or low-energy electron (LEE) induced DNA damage [[79], [80], [81]]. Here, biotinylated DNA species were presented in defined patterns on the surface of a DNA origami, readily identifiable by AFM with the addition of streptavidin. Following selective radiation exposure, the absolute cross-section of DNA strand breakages was directly quantified by the resultant streptavidin patterns atop the DNA origami. This elegant approach was used to assess the radiation susceptibility of G-quadruplex-rich telomeric DNA regions [82], the impact on specific DNA sequences [83] and the effects of several clinically relevant radiotherapy photosensitisers [84] [85]. This concept can be expanded through the introduction of DNA aptamers to remove the limitation of detection *via* hybridisation or damage of nucleic acids. As such, these systems are now capable of assaying proteins and small molecules, which was first demonstrated for Thrombin on triple helical DNA tiles [86] and later on DNA origami [87]. The use of DNA aptamers precludes the need for additional chemistry or the use of bulky binding agents – such as antibodies – meaning that appendages of the DNA nanostructure itself can directly contain the binding sequences, simplifying the system to a one-pot reaction. Moreover, indirect reporting of enzyme activity can also be achieved, where their action upon DNA – *e.g.* methylation – restores the structure of the aptamer enabling binding of a reporter [88].

Using this combination of DNA aptamer and DNA nanostructure, Rinker et al. [89]. demonstrated the distance-dependent binding ability of a pair of thrombin aptamers. Here, binder spacing was optimised at 5.8 nm, attaching to either side of the thrombin protein. Larger separations were shown to result in only single binder interactions which significantly reduced affinity and therefore the efficiency of the assay. Recently, the energetics of this system have been probed independently by others [90]. By simulating a dual aptamer platform the authors provide evidence to suggest that parallel binder approaches provide the most robust routes to biosensing with improved measurement confidence and reduced crosstalk. This work highlights the advantage of the spatial addressability that DNA nanostructures can provide within bio-sensing and bio-labelling applications. In order to ensure detection confidence and reduce non-specific binding producing false positives, it is preferable to have a two-pronged approach of binding multiple analytes or binding the same analyte with multiple probes simultaneously. However, typically the latter approach is hampered by the random distribution of the binder pairs when attached to support surfaces using bulk chemistry. Here, DNA nanostructures are suitably placed to act as an adapter between the support surface whilst providing spatial precision to binding pair placements. This has been expertly applied to the development of platforms which have helped to define the spatial binding tolerance of antibodies [91] and for fragment-based drug discovery [92].

Furthermore, DNA nanostructure-based biosensors have been demonstrated within complex biological fluids, where Mei et al. [93]. achieved a detection limit of 15 nM for a thrombin target directly from spiked cell lysate. Additional examples include the work of Godonoga *et al* [94] where 12 DNA aptamers specific to *Plasmodium falciparum* lactate dehydrogenase (PfLDH) – a malarial infection marker – were arrayed on the surface of a rectangular DNA nanostructure enabling its detection down to 500 nM within blood plasma. In the latter work, binding of the marker to the functionalised DNA nanostructure was confirmed in real-time using HS-AFM, but was also sampled indirectly as the PfLDH was shown to retain its biochemical activity when bound. These studies continue to broaden the applicability of DNA nanostructures in biosensing, however, there remains some way to go before the wide-spread adoption of this technology. Interestingly, the approach using DNA nanostructures for biosensing can also be inverted, and aptamer-labelled DNA nanostructures can be used for targeted delivery or response. For example the targeted release of a chemotherapy agent upon binding to tumour-specific markers on a cell surface has been demonstrated [95,96].

**Fig. 6:**
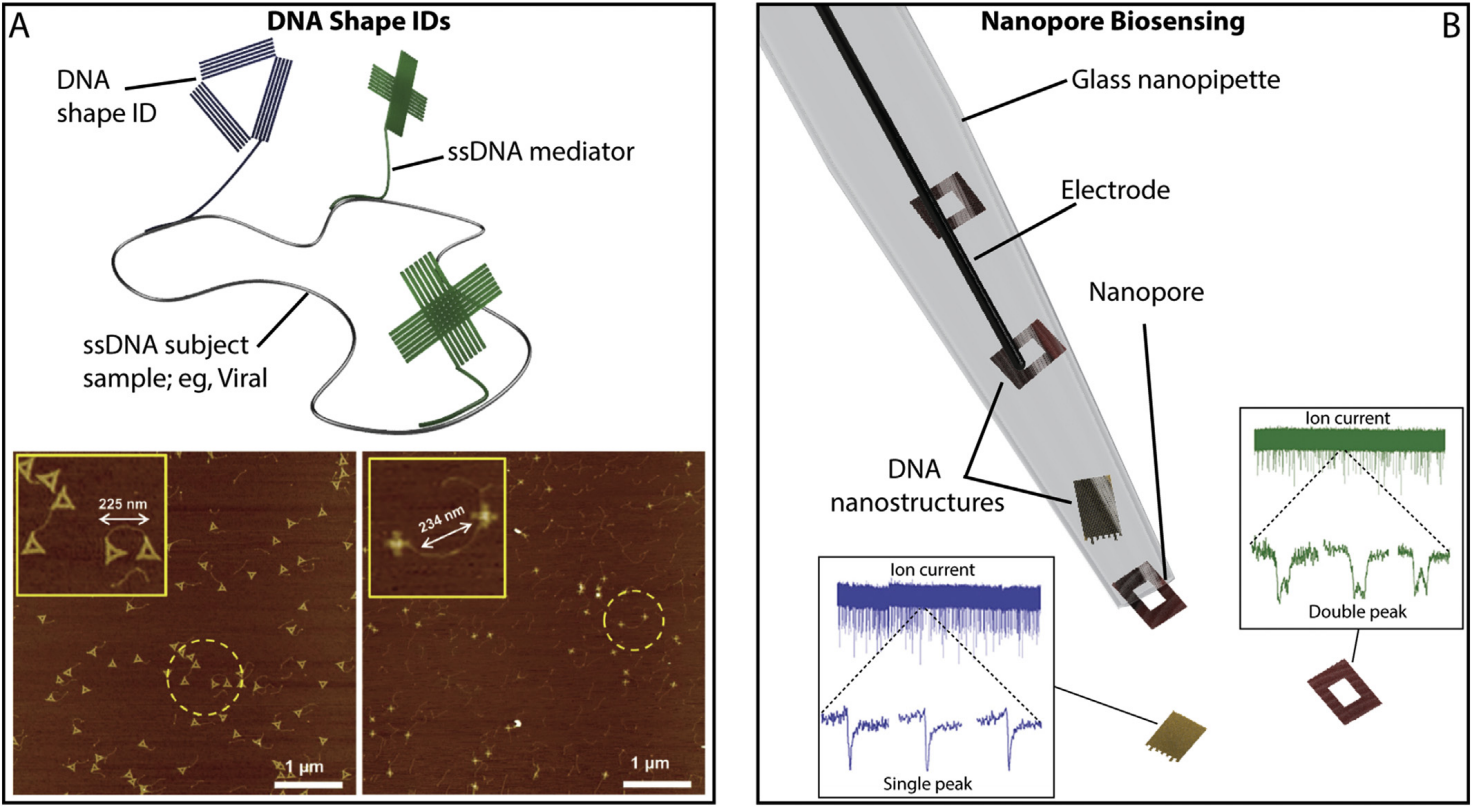
DNA-nanostructure based biosensing. Alongside the co-localisation of biological molecules in sensing applications, the DNA nanostructures have been used for signal amplification. A) The structure of the DNA construct is used to provide a unique visual cue to the presence or absence of specific sequences in a DNA sample through the hydridisation of mediator strands. B) Translocation of DNA nanostructures through a nanopore provides a unique ion current profile, which can be used to identify a particular DNA nanostructure. A and B are adapted with permission from Liu et al [97] copyright: (2018) John Wiley and Sons, and Raveendran et al [6] copyright: (2018) John Wiley and Sons, respectively.

Thus far, with few exceptions, the sensing mechanisms of these systems relies on the direct observation of the analyte bound to the designed locations on the DNA nanostructure. This approach precludes the detection of analytes which are too small to be reliably identified by AFM. In response, alternative signal amplification approaches have been demonstrated, such as the gold nanoparticle-coupled DNA probe NOT-gate work by Lu et al. [98]. or the DNA-based nanomechanical tweezers presented by Kuzuya et al. [99]. In the latter, the binding of the analyte results in a conformational change in the DNA device from an open to closed configuration. This system was shown to amplify the detection of a variety of small analytes, in addition to reporting on buffer composition and enzyme activity [100]. An analogous approach by Wang and co-workers [101] utilised the shape of the DNA nanostructures themselves as the specific detection marker, demonstrating the detection of SNPs within disease-associated genes or directly genotyping Hepatitis B viruses (Fig. 6A) [97]. Here, several DNA-based shapes, including a triangle and cross, were hybridised directly to genomic DNA substrates *via* ‘mediator’ strands which were then used to identify visually – by AFM – particular regions in the genome sample and even discriminate between sequences with single base resolution [101]. An alternative to AFM-based detection approaches has recently been demonstrated by us, where the DNA nanostructures were shown to produce characteristic ion current peaks for different shapes as a result of their translocation through the pore of a nanopipette providing a novel method of structural fingerprinting (Fig. 6B) [6]. In contrast, an interesting recent development by Torelli *et al* [102] demonstrated an actuatable enzyme encapsulating DNA nanostructure tethered to the end of an optical fibre in order to provide an analyte-responsive localised chemiluminescent sensor [102]. Furthermore, others have demonstrated plasmonic detection approaches, including the use of DNA origami as a spatial adapter between the binding agents and the optical fibres for surface plasmon resonance (SPR), [103] and the development of a switchable chiral plasmonic DNA-device [104].

## 7 Outlook

DNA nanotechnology offers a unique and versatile toolset to localise and host single biological entities as well as multicomponent pathways. These tools confer many advantages over the more contemporary approaches and enable the studying of living systems at the relevant length scales – including straight-forward scalability, chemical addressability, ability to place molecules of interest with exquisite sub-nanometre resolutions, perform responsive functions and even undertake logic operations.

To date, an array of tools has been demonstrated which, amongst others, augment microscopic observations, probe biomolecule mechanics, and detect analytes from complex biological fluids. However, the field is far from being mature or saturated, with many of the described approaches only in their infancy.

Physiologically, enzymes rarely work alone, and their action is typically the culmination of a network of interactions which tightly regulates their biological functionality. Arguably one of the greatest contributions of DNA nanotechnology within this area is that it offers the potential to reconstitute entire biological pathways, thereby providing an approach to address spatially multi-component complexes and networks, beyond the two enzyme systems demonstrated to date. This is a very achievable goal and one which can be considered primarily as a challenge of scalability of the current DNA nanotechnology tools to address larger complexes rather than isolated enzymes. An example of this is the dissection of ribosome assembly, where a scalable DNA nanostructure would enable the characterisation of the hierarchical assembly of ribosome subunits to be studied in real time. This would provide insight into how and at what stage the collective function of the subunits begin to arise and how their global function is orchestrated.

Building modular DNA-based systems in this way would allow enzyme cascades to be presented in full and dissected into their constituent interactions within the same environmental context. Moreover, the internal organisation of these pathways could be subsequently manipulated to engineer synthetic alternatives, offering enhanced efficiencies or to generate a non-native biochemistry, such as the development of a synthetic light harvesting complex. Where this is applied to closed systems – for example those bound by membranes – routes towards artificial protocells may emerge. This is arguably one of the great challenges of biological study, enabling the recapitulation of basic physiological units step-by-step to build up complexity and to investigate the minimum requirements of life. Indeed, DNA nanotechnology has already made some impressive contributions in this endeavour to date, including the development of DNA-templated membrane-spanning nanopores, [105] DNA structures for membrane remodelling, [106] and a DNA-based nuclear pore complex (NPC) including the integral array of intrinsically disordered proteins, [107] to name but a few.

Moreover, continued progress in the related field of DNA computing has made significant strides in recent years, with ever more complex DNA reaction networks being demonstrated (an excellent review is given by Fu et al.) [108]. These systems are now capable of providing basic logic functions to autonomous protocells and have even been shown to conduct intercellular DNA-based communications within populations [109]. As such, it is conceivable that in the future we will be able to reconstitute many biological functions within artificial systems, constructed from and programmed by DNA. Indeed, systems designed from DNA are now emerging which are capable of detecting a specific analyte, conducting logic-based functions and providing the targeted delivery of a therapeutic payload in response [96].

With this in mind, it is clear that DNA nanotechnology has a transformative effect on our understanding of biological processes, which in turn will enable us to address many clinical challenges through the application of nanostructured diagnostics assay systems, responsive biosensors, contextualised microscopic imaging and specifically targeted or even personalised therapeutics.

## Funding

Engineering and Physical Sciences Research Council Centre for Doctoral Training in Molecular Scale Engineering [EP/J500124].

## Data Availability

Data supporting this work can be accessed *via* the University of Leeds repository: https://doi.org/10.5518/612

## Declarations of Competing interest

The authors declare no conflict of interest.

## Acknowledgements

A.J.L. would like to acknowledge the support of the EPSRC CDT in Molecular Scale Engineering.
